# 515. Neurological Complications in Children Hospitalized for Influenza, 2010 to 2022, the Canadian Immunization Monitoring Program Active (IMPACT)

**DOI:** 10.1093/ofid/ofae631.167

**Published:** 2025-01-29

**Authors:** Esther Vaugon, Shaun Morris, Julie A Bettinger, Catherine Burton, Scott Halperin, Taj Jadavji, Kescha Kazmi, Jacqueline Modler, Manish Sadarangani, Jesse Papenburg

**Affiliations:** Centre Hospitalier Universitaire Sainte-Justine, Université de Montréal, Montreal, QC, Canada; Hospital for Sick Children, University of Toronto, Toronto, Ontario, Canada; University of British Columbia, Vancouver, British Columbia, Canada; University of Alberta, Edmonton, AB, Canada; IWK Hlth Ctr, Halifax, NS, Canada; Alberta Children’s Hospital, University of Calgary, Calgary, Alberta, Canada; Hospital for Sick Children, University of Toronto, Toronto, Ontario, Canada; Research Institute of the McGill University Health Centre, Montreal, Quebec, Canada; University of British Columbia, Vancouver, British Columbia, Canada; McGill University Faculty of Medicine and Health Sciences, Montreal, Quebec, Canada

## Abstract

**Background:**

Influenza-associated neurological complications are rare, but can cause significant morbidity and mortality. We aimed to estimate the proportion of children with neurological complications among those hospitalized for influenza and to identify associated risk factors. The secondary objective was to compare the risk of severe outcomes among children with neurological complications to that of those without.

**Methods:**

Prospective active surveillance was conducted in 12 pediatric hospitals from the Immunization Monitoring Program, ACTive (IMPACT), representing >90% of all tertiary pediatric beds in Canada. We included patients aged ≤ 16 years hospitalized for microbiologically confirmed influenza during the 2010-11 to 2021-22 influenza seasons. Regression analyses were used to calculate adjusted odds ratios (aORs) for factors associated with neurological complications (seizure, meningitis, encephalitis, myelitis, encephalopathy, radiculopathy, cerebral edema, hydrocephalus, or stroke). The risk of severe outcomes (intensive care unit admission, mechanical ventilation, or death) in children with neurological complications was compared to that of children without these complications using the Chi-square and Wilcoxon tests.

**Results:**

Among the 9533 children hospitalized for influenza, 1228 (12.9%) had neurological complications. Children with neurological complications had longer hospitalization (6.3 days vs 4.5 days, p < 0.001) and were at higher risk of intensive care unit admission (31.8% vs 14.1%, p < 0.001), mechanical ventilation (20.5% vs 4.4%, p < 0.001), and death (2.2% vs 0.3%, p < 0.001). In a multivariable model adjusting for IMPACT site and influenza type, factors associated with neurological complications were: pre-existing neurological condition (aOR 4.19, 95% CI 3.61-4.86); age between 6 and 24 months (aOR 1.62, 95% CI 1.29-2.03) and age between 2 and 4 years (aOR 1.36, 95% CI 1.09-1.71).

**Conclusion:**

More than one in ten children hospitalized for influenza developed neurological complications and these were associated with higher risk of severe outcomes. Risk factors identified for neurological complications were age between 6 months and 4 years and the presence of a pre-existing neurological condition.

**Disclosures:**

**Shaun Morris, MD, MPH, DTM&H, FRCPC, FAAP**, GSK Canada: Advisor/Consultant|GSK Canada: Honoraria|Pfizer: Advisor/Consultant|Pfizer: Honoraria|Sanofi Canada: Advisor/Consultant|Sanofi Canada: Honoraria **Jesse Papenburg, MD, MSc**, Merck: Honoraria|Merck: Site investigator, RCT

